# TGF-β1 Receptor Inhibitor SB525334 Attenuates the Epithelial to Mesenchymal Transition of Peritoneal Mesothelial Cells via the TGF-β1 Signaling Pathway

**DOI:** 10.3390/biomedicines9070839

**Published:** 2021-07-19

**Authors:** Jung-Yoon Heo, Jun-Young Do, Yunmee Lho, A-Young Kim, Sang-Woon Kim, Seok-Hui Kang

**Affiliations:** 1 Department of Internal Medicine, Smart-Aging Convergence Research Center, College of Medicine, Yeungnam University, Daegu 42415, Korea; yuni0325@ynu.ac.kr (J.-Y.H.); ckdwjdgus@naver.com (Y.L.); 2 Division of Nephrology, Department of Internal Medicine, College of Medicine, Yeungnam University, Daegu 42415, Korea; jydo@med.yu.ac.kr (J.-Y.D.); dkdud0904@naver.com (A.-Y.K.); 3 Division of Gastro-Enterology, Department of Surgery, College of Medicine, Yeungnam University, Daegu 42415, Korea; swkim@med.yu.ac.kr

**Keywords:** SB525334, epithelial mesenchymal transition, transforming growth factor-beta1, peritoneal fibrosis, peritoneal mesothelial cell

## Abstract

We investigated the effect of SB525334 (TGF-β receptor type 1 (TβRI) inhibitor) on the epithelial to mesenchymal transition (EMT) signaling pathway in human peritoneal mesothelial cells (HPMCs) and a peritoneal fibrosis mouse model. In vitro experiments were performed using HPMCs. HPMCs were treated with TGF-β1 and/or SB525334. In vivo experiments were conducted with male C57/BL6 mice. The 0.1% chlorhexidine gluconate (CG) was intraperitoneally injected with or without SB52534 administration by oral gavage. Mice were euthanized after 28 days. EMT using TGF-β1-treated HPMCs included morphological changes, cell migration and invasion, EMT markers and collagen synthesis. These pathological changes were reversed by co-treatment with SB525334. CG injection was associated with an increase in peritoneal fibrosis and thickness, which functionally resulted in an increase in the glucose absorption via peritoneum. Co-treatment with SB525334 attenuated these changes. The levels of EMT protein markers and immunohistochemical staining for fibrosis showed similar trends. Immunofluorescence staining for EMT markers showed induction of transformed cells with both epithelial and mesenchymal cell markers, which decreased upon co-treatment with SB525334. SB525334 effectively attenuated the TGF-β1-induced EMT in HPMCs. Cotreatment with SB525334 improved peritoneal thickness and fibrosis and recovered peritoneal membrane function in a peritoneal fibrosis mouse model.

## 1. Introduction

Chronic kidney disease (CKD) is an increasingly prominent health problem and can progress to end-stage renal disease, which requires renal replacement therapy. Peritoneal dialysis (PD) has been one of the most important dialysis modalities in treating CKD since the first PD trial by Ganter, in 1923 [1]. PD requires peritoneal dialysate within the peritoneal cavity for solute transport or water removal. Artificial peritoneal dialysate lacks biocompatibility, owing to factors such as high glucose, osmolarity, or glucose degradation products, which results in peritoneal membrane damage [2,3]. Repeated peritoneal membrane damage can lead to fibrosis and/or thickening of the peritoneal membrane. These pathological changes can ultimately lead to peritoneal membrane failure and inability to properly remove uremic toxins or water. Furthermore, excessive fibrosis can lead to encapsulating peritoneal sclerosis, resulting in high mortality and morbidity [4]. Therefore, proper prevention, attenuation, or interventions is necessary to maintain long-term PD and improve the prognosis of patients with PD.

The transforming growth factor-beta 1 (TGF-β)-induced epithelial to mesenchymal transition (EMT) plays a key role in the development of PD-induced peritoneal fibrosis (PF) [2,3]. TGF-β binds to latency-associated peptides stored in the extracellular matrix and is released by various stress stimuli [5]. Active TGF-β binds to TGF-β receptor type 2 (TβRII), which phosphorylates the serine and threonine residues on TGF-β receptor type 1 (TβRI), thereby inducing the phosphorylation of serines on Smad 2 and 3 [6,7]. Consequently, the transcription of the TGF-β-target genes, such as Snail, is initiated, which, in turn, triggers EMT. SB525334 is a potent TβRI inhibitor; previous studies have demonstrated that SB525334 blocks TGF-β-induced Smad activation and decreases EMT in some tissues/cell lines [8,9,10,11]. Considering the association between TGF-β signaling and EMT in peritoneal mesothelial cells, SB525334 may attenuate TGF-β signaling, resulting in a decrease in EMT and peritoneal membrane fibrosis. In this study, we investigated the effect of SB525334 on the EMT signaling pathway in human peritoneal mesothelial cells (HPMCs) and further examined its influence on the peritoneal membrane in a PF mouse model.

## 2. Materials and Methods

### 2.1. HPMC Culture and Treatment Conditions

The Institutional Review Board of Yeungnam University Medical Center (IRB No: 2019-04-047 at 21 December 2019) approved HPMC collection using human specimens. Briefly, gastrectomy was considered for all biopsy-confirmed patients with stomach cancer and was performed using partial or total gastrectomy with total or partial omentectomy. The remnant omentum is typically discarded at our center after surgery. Therefore, prior to surgery, patient informed consent was obtained for use of mesothelial cells from the discarded omentum. HPMCs were isolated as previously described [12,13,14]. In vitro experiments were performed on cells after one to two passages. HPMCs were cultured in M199 medium supplemented with L-glutamine (20 μM, Gibco, Waltham, MA, USA), penicillin-streptomycin (150 units or 150 μg/mL, Gibco), hydrocortisone (0.4 μg/mL, Sigma-Aldrich, St. Louis, MO, USA), apotransferrin (5 μg/mL, Sigma), insulin (5 μg/mL. Sigma) and 10% fetal bovine serum (FBS) (Gibco). Cell cultures were maintained in 100 mm dishes (Nunc, Rochester, NY, USA) in a 5% CO_2_ humidified atmosphere incubator (311 Forma Direct Heat CO_2_ incubator, Thermo Fisher Scientific, Waltham, MA, USA) at 37 °C.

### 2.2. Cytotoxicity Assay

The cytotoxicity of SB525334 (S1476, Selleckchem, Houston, TX, USA) was evaluated using the MTT assay (M6494, Invitrogen, Waltham, MA, USA). Approximately, 10^4^ HPMCs/well were initially seeded and cultured in 96-well plates (0494, Corning, NY, USA) for 24 h, then treated with SB525334 (0 μM, 0.1 μM, 1 μM, 1 μM, 10 μM, 100 μM and 200 μM) in 200 μL media for 24–72 h. At the end of the incubation period, 20 μL of MTT (5 mg/mL) was added to each well and incubated at 37 °C for 3.5 h. Subsequently, 200 μL of DMSO was added to each well to solubilize the formazan products. Absorbance was measured at 570 nm using a microplate reader (Synergy HTX, BioTek Instruments Inc., Winooski, VT, USA).

### 2.3. Cell Morphology, Wound Healing and Invasion Tests

HPMCs in culture dishes were incubated in a M199 culture medium for 24 h for cell cycle synchronization. The growth medium was then replaced with serum-free M199 medium supplemented with 1% FBS and 2.0 ng/mL of TGF-β (R&D Systems, Minneapolis, MN, USA), with or without SB525334 (0.1 μM, 1 μM, 2 μM, 5 μM, or 10 μM), for 24–72 h. Cell morphology was analyzed using an inverted phase-contrast microscope (200×) (DMi8, Leica, Wetzlar, Germany).

A wound healing/migration assay was used to detect cell migration. HPMCs were seeded at a density of 10^5^ cells/well in a culture-insert-2 well (81176, Ibidi GmbH, Martinsried, Germany). After allowing the cells to attach overnight, the culture insert was removed and the cells were washed with phosphate-buffered saline (PBS) (PR2007-100-00, Biosesang, Seongnam, Korea) to remove nonadherent cells. The growth medium was then replaced with a fresh serum-free M199 medium, supplemented with 1% FBS and TGF-β (2.0 ng/mL), with or without SB525334 (1 μM), and the plate was photographed at 0 h and 16 h. The number of cells migrating to the wound area was manually counted (in three fields per well) under a light microscope at 50× magnification [15].

The cell invasion assay was performed using Transwell inserts with 8 µm pore size polycarbonate membranes (3422, Corning), according to a previously described protocol with slight modifications [16]. Briefly, the inner surface of the membrane was coated with 20 µL of Matrigel (0.5 mg/mL, 354234, Corning) and incubated overnight at 37 °C to solidify the Matrigel. The outer surface was coated with 30 µL of type I collagen (0.5 mg/mL, 354236, Corning) and incubated for 3 h at 37 °C. After drying, 100 µL of serum-free M199 medium, supplemented with 2% FBS and 700 µL of serum-free M199 medium, containing 10% FBS, was added to the upper and lower chambers of the Transwell system, respectively. After conventional digestion, the cells in each group were resuspended in serum-free M199 medium at a density of 10^5^ cells/flask. A total of 200 µL of cell suspension was added to the upper chamber of the Transwell system on top of the Matrigel coating. After incubation at 37 °C for 16 h, the invaded cells were sequentially fixed in 4% paraformaldehyde (pc2031-100-00, Biosesang) for 10 min, methanol for 5 min and 0.5% crystal violet solution (V5265, Sigma-Aldrich), prepared in methanol, for 1 h. Stained cells were counted using a light microscope (100×).

### 2.4. Western Blotting

The primary antibodies against E-cadherin (610181, BD Biosciences, Franklin Lakes, NJ, USA), α-SMA (α-smooth muscle actin, A2547, Sigma-Aldrich), Snail (#3895, Cell Signaling Technology, Boston, MA, USA) (all 1:1000) and the corresponding horseradish peroxidase (HRP)-conjugated secondary anti-mouse IgG (sc-516102, Santa Cruz Biotechnology, Dallas, TX, USA) (1:2000) and the primary antibodies against fibronectin (ab268020, Abcam, Cambridge, MA, USA), GAPDH (#2118, Cell Signaling Technology), phospho-Smad2 (#3108, Cell signaling Technology), phospho-Smad3 (#9520, Cell Signaling Technology) and Smad2/3 (#3102, Cell signaling Technology) (all 1:1000) and the corresponding HRP-conjugated secondary anti-rabbit IgG (A16096, Invitrogen) (1:2000) were diluted at indicated concentrations.

The harvested HPMCs and tissues were subjected to 10% SDS-PAGE on appropriate resolving gels and immunoblotted. Briefly, tissues and cells were lysed in ice-cold RIPA buffer (25 mM Tris-HCl (pH of 7.6), 150 mM NaCl, 1% NP-40, 1% sodium deoxycholate, 0.1% SDS (89901, Thermo Fisher Scientific)), containing 1% protease inhibitor single-use cocktail solution (100×, 1 mM AEBSF, 800 nM aprotinin, 50 μM bestatin, 15 μM E64, 20 μM leupeptin, 10 μM pepstatin A and 5 mM EDTA (78430, Thermo Fisher Scientific)). The lysates were centrifuged at 14,000 rpm for 10 min at 4 °C and the supernatants were collected. Proteins were separated with 10% SDS-PAGE and transferred onto 0.45 μm PVDF membranes (GE10600023, Amersham, GE Healthcare, Buckinghamshire, UK). The membranes were blocked with 5% skim milk (MB-S1667, MBcell, Seoul, Korea) in TBS-T (247 mM Tris (pH of 7.4), 1.37 M NaCl, 27 mM LCl and 0.5% Tween 20 (TR2007-100-74, Biosesang)), before incubation overnight at 4 °C with the primary antibodies. Next, the membranes were washed with TBS-T and incubated with appropriate HRP-conjugated secondary antibodies. Protein bands were detected using enhanced chemiluminescence reagents (34095, Thermo Fisher Scientific). The membranes were assessed using LAS-3000 (Fujifilm, Tokyo, Japan) and the density of the area was evaluated using the ImageJ software (version 1.50e, National Institutes of Health, Bethesda, MD, USA).

### 2.5. Immunofluorescence Staining

HPMCs (2 × 10^5^ cells per chamber) were grown on an 8-well chamber slide (154534, Thermo Fisher Scientific) for 24 h for cell cycle synchronization. The growth medium was then replaced with serum-free M199 medium supplemented with 1% FBS and TGF-β (2.0 ng/mL), with or without SB525344 (1 μM), for 24 h. For immunofluorescence staining, cells were washed in 1% bovine serum albumin (BSA) (160069, MP Biomedicals, Illkirch, France) dissolved in PBS and fixed in 4% paraformaldehyde (10 min at 4 °C). They were then permeabilized with 0.1% Triton X-100 in PBS (15 min at 4 °C) and washed again with 1% BSA in PBS. After an overnight incubation at 4 °C with primary antibodies against E-cadherin (610181, BD biosciences, Franklin Lakes, NJ, USA) (1:100), α-SMA (#19245, Cell Signaling Technology) (1:200) and COL1A1 (collagen type 1 alpha 1, #72026, Cell Signaling Technology) (1:500), the cells were treated with 1% BSA in PBS for 1 h and, subsequently, washed again with PBS. The cells were then incubated for 1 h with a fluorescein-conjugated secondary antibody, either Alexa Fluor 488-conjugated goat anti-mouse IgG (A11001, Invitrogen), or Alexa Fluor 568 goat anti-rabbit IgG (A11012, Invitrogen). A slide chamber was mounted using mounting medium containing DAPI (H-1200, Vector Laboratories, Burlingame, CA, USA), in the dark at room temperature. Nuclei were counterstained with DAPI and the stained slide chamber was examined under a light microscope (400×).

### 2.6. Quantitative Reverse Transcriptase Polymerase Chain Reaction (qRT-PCR) Analysis

qRT-PCR was performed in a 20 μL reaction mixture containing 10 μL of iQ™ SYBR^®^ Green Supermix (1708880AP, Bio-Rad, Hercules, CA, USA), 1 μL each of 10 pmole/μL forward and reverse primers, 6 μL of water and 2 μL of template cDNA. The primer sequences used were as follows: COL1A1 forward, 5′-GCCTCAAGGTATTGCTGGAC-3′, reverse, 5′-ACCTTGT TTGCCAGGTTCAC-3′; Snail forward, 5′-GTTTACCTTCCAGCAGCCCT-3′, reverse, 5′-TCCCAGATGAGC ATTGGCAG-3′; β-actin forward, 5′-ATCGTGCGTGACATTAAGGA-3′, reverse 5′-ATTGCCAATGGTGATGACCTG-3′. Relative mRNA expression levels of the target genes in each sample were calculated using the comparative CT method. The relative expression of each gene was normalized to that of β-actin. The samples were assayed on a CFX Connect real-time System (CFX Connect Optics Module, Bio-Rad).

### 2.7. Animal Experiments

All experiments were conducted with male C57/BL6 mice (10 weeks, 20–25 g) (Samtako Biokorea, Seoul, Korea). Mice were group-housed under a 12:12 h light:dark cycle at 24 ± 1 °C. Mice had unrestricted access to standard tap water and were allowed to acclimatize to the environment for at least seven days. All animal procedures were approved by the Institutional Review Board of Yeungnam University College of Medicine (IRB No: YUMC-AEC-2019-026 at 12 August 2020) and were in accordance with the Guide for the Care and Use of Laboratory Animals.

PF was induced using a previously described protocol, with slight modification [17]. Mice were classified into three groups as follows: (i) CTL (*n* = 5), 15% ethanol dissolved in PBS (1.5 mL/100 g body weight) was intraperitoneally injected every other day and oral gavage with corn oil (0.1 mL/body, C8267, Sigma-Aldrich) containing 0.5% DMSO was performed daily; (ii) PF (*n* = 6), 0.1% chlorhexidine gluconate (CG) and 15% ethanol dissolved in PBS (1.5 mL/100 g body weight) were intraperitoneally injected every other day and oral gavage with corn oil (0.1 mL/body) containing 0.5% DMSO was performed daily; (iii) PF + SB (*n* = 8), 0.1% CG and 15% ethanol dissolved in PBS (1.5 mL/100 g body weight) were intraperitoneally injected every other day and oral gavage with SB52534 (20 mg/kg/day), including corn oil and 0.5% DMSO, was performed daily. To avoid artifacts arising from peritoneal damage due to repeated injections, 0.1% CG was injected at the lower part of the peritoneum and the upper portion of the parietal peritoneum was used for the analyses. Mice were euthanized after 28 days. All mice were anesthetized via intraperitoneal injection of a combination of tiletamine (125 μg/g), zolazepam (125 μg/g) (Virbac, Seoul, Korea) and xylazine (0.78 μg/g) (260, Bayer-Korea, Seoul, Korea).

### 2.8. Peritoneal Equilibration Test

Mice in each group underwent a peritoneal equilibrium test on the day before euthanasia. Mice were injected with 2 mL of 4.25% glucose solution containing the dialysate (Physioneal^®^, Baxter Healthcare, Singapore) for 2 h and euthanized; then, the dialysate was collected. Glucose levels in the dialysate were determined according to the manufacturer’s instructions (Seoul Clinical Laboratories, Seoul, Korea). Functional alteration of peritoneal membranes was evaluated by D/D0 glucose levels at 2 h after dialysate infusion per level at 0 h [18].

### 2.9. Histological Analysis of the Peritoneum

The parietal peritoneum of the abdominal wall or visceral peritoneum of the liver surface was fixed with 4% paraformaldehyde, embedded in paraffin and cut into 4 μm thick sections. Thickness of the parietal peritoneum, including the mesothelium and submesothelial tissue, was measured in tissue sections under a light microscope (100×) after staining with Masson’s trichrome (Trichrome stain kit, TRM-2, ScyTek, Logan, UT, USA). Slide scans were performed using a panoramic digital slide scanner (Panoramic scan2, 3Dhistech Ltd., Budapest, Hungary) and the thickness was measured using the Caseviewer software (3Dhistech Ltd.).

For immunohistochemical staining, tissues were fixed with 4% paraformaldehyde (pH of 7.4), embedded in paraffin and cut into 4 μm sections using a microtome. Tissue sections were rehydrated using xylene and an ethanol gradient. After washing with water for 5 min, the sections were permeabilized using 3% H_2_O_2_ (1145, Duksan, Ansan, Korea), dissolved in methanol, for 15 min, then washed with water twice for 5 min. For antigen unmasking, sections were immersed in citrate buffer (150 mM sodium citrate, pH of 6.0), boiled for 10 min, cooled at room temperature for 20 min, washed with water and blocked with 5% normal goat serum blocking solution (NGS) (S-1000-20, Vector Laboratories) in PBS for 30 min. The sectioned tissues were incubated with anti-collagen I (GTX 20292, GeneTex, Irvine, CA, USA) (1:200), anti-fibronectin (ab268080, Abcam) (1:200), or anti-TGF-β antibodies (ab215715, Abcam) (1:200), dissolved in 5% NGS, overnight at 4 °C. The sections were then washed with PBS, followed by incubation with a goat anti-rabbit antibody (A16096, Invitrogen) (1:200), dissolved in 5% NGS, at room temperature for 1 h. Subsequently, the sections were washed with PBS for 10 min, stained according to the manufacturer’s instructions (DAB Substrate Kit for Peroxidase, SK-4105, Vector Laboratories) and counterstained with hematoxylin (S3309, DAKO, Carpinteria, CA, USA). Sections were then dehydrated using an ethanol gradient, cleared in xylene and covered with cover slips using a mounting medium solution (3801120, Leica). For immunofluorescence microscopy, 15 μm tissue sections were incubated and processed in the same manner as the in vitro samples.

### 2.10. Statistical Analysis

IBM SPSS Statistics (version 25.0, IBM Corp., Armonk, NY, USA) was used to analyze the data. Data are expressed mean ± standard error. Groups were compared using the Kruskal–Wallis or Mann–Whitney U rank-sum test. Differences between time points were compared using the Wilcoxon signed-rank test. The association between two continuous variables was evaluated using a linear regression analysis. Statistical significance was set at *p* < 0.05.

## 3. Results

### 3.1. SB525334 Attenuates Morphological Changes and Cell Migration in TGF-β-Treated HPMCs

**Figure 1:**
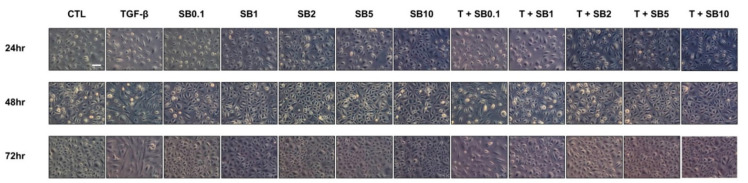
Effect of SB525334 on TGF-β-induced morphological changes (200× magnification; scale bar, 100 μm). The cobblestone appearance of HPMCs transformed to spindle-shaped upon TGF-β treatment. Morphological changes were attenuated by co-treatment with SB525334. CTL, control; TGF-β, transforming growth factor-beta 1; SB0.1, cells treated with 0.1 μM SB525334; SB1, cells treated with 1 μM SB525334; SB2, cells treated with 2 μM SB525334; SB5, cells treated with 5 μM SB525334; SB10, cells treated with 10 μM SB525334; T + SB0.1, SB0.1 with TGF-β; T + SB1, SB1 with TGF-β; T + SB2, SB2 with TGF-β; T + SB5, SB5 with TGF-β; T + SB10, SB10 with TGF-β.

Supplementary Figure S1 shows the results of SB525334 toxicity according to the treatment duration. Cell toxicity was observed after 24 h of treatment with 100 μM SB525334. Morphological changes from cobblestone to spindle-shape were observed 24 h after TGF-β administration and worsened over time. SB525334 alone did not result in morphological changes and TGF-β-induced morphological changes were attenuated at SB525334 levels above 1 μM (Figure 1).

**Figure 2:**
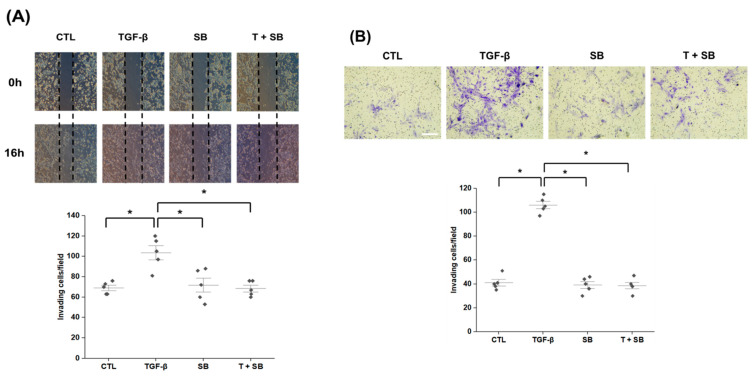
Effect of SB525334 on TGF-β-induced cell migration and invasion. (**A**) Wound healing assay showing TGF-β-induced wound closure attenuated by co-treatment with SB525334 (1 μM, 50× magnification). (**B**) Matrigel invasion assay showing TGF-β-induced invasion attenuated by co-treatment with SB525334 (100× magnification; scale bar, 200 μm). CTL, control; TGF-β, transforming growth factor-beta 1; SB, cells treated with 1 μM SB525334; T + SB, SB with TGF-β. Quantification of invading cells is expressed as mean ± standard error (*n* = 5 per group). * *p* < 0.05, compared to the corresponding group.

Wound healing and Matrigel tests showed that cell migration and invasion were more prominent in TGF-β-treated HPMCs; however, SB525334 attenuated these effects (Figure 2).

### 3.2. Effects of SB525334 on EMT Markers in TGF-β-Treated HPMCs

**Figure 3:**
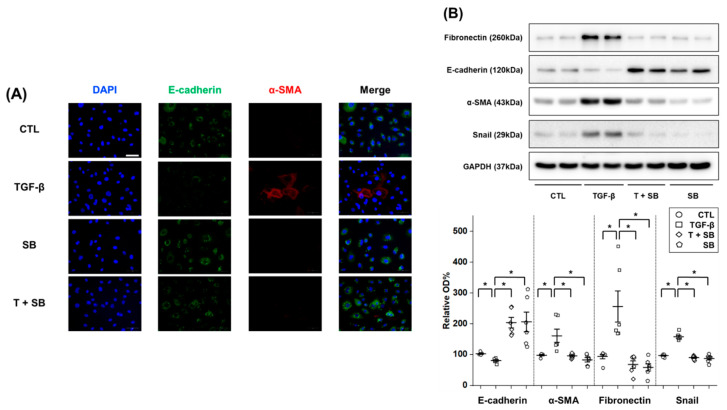

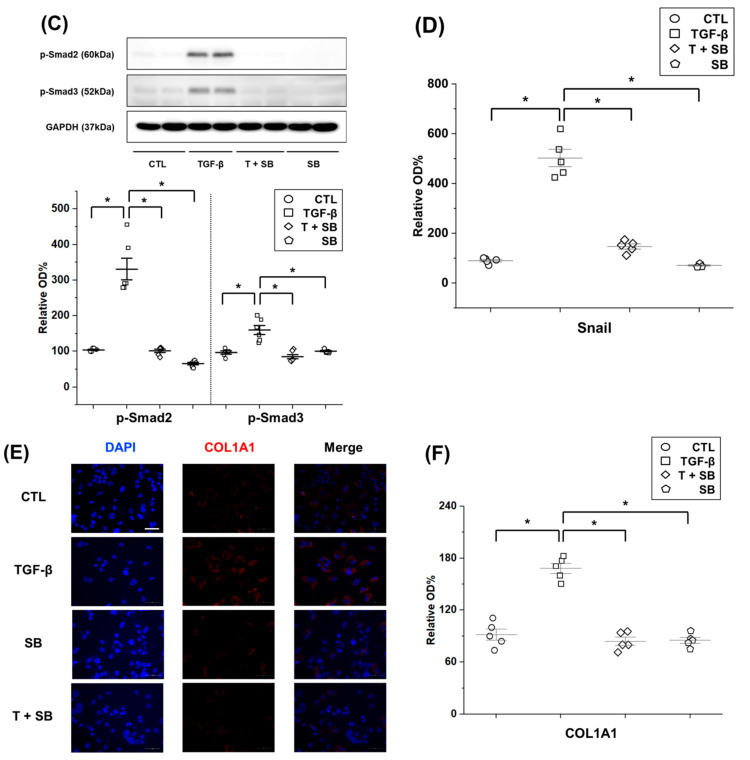
Changes in epithelial and mesenchymal cell markers in TGF-β-treated-human peritoneal mesothelial cells, with or without SB525334 (1 μM). (**A**) Immunofluorescence staining of E-cadherin (green) and α-SMA (red) with nuclear counterstaining (DAPI; blue) (400× magnification; scale bar, 50 μm). Western blotting results for (**B**) epithelial and mesenchymal cell markers and (**C**) the Smad-dependent signaling pathway. (**D**) mRNA expression of Snail. (**E**) Immunofluorescence staining of COL1A1 (red) with nuclear counterstaining (DAPI; blue) (400× magnification; scale bar, 50 μm). (**F**) mRNA expression of COL1A1. CTL, control; TGF-β, transforming growth factor-beta 1; SB, cells treated with 1 μM SB525334; T + SB, SB with TGF-β; α-SMA, α-smooth muscle actin; OD, optical density; p-Smad2, phospho-Smad2; p-Smad3, phospho-Smad3; COL1A1, collagen type 1 alpha 1. Percentage for the control group is expressed as mean ± standard error (*n* = 6 per group for protein level and *n* = 5 per group for mRNA level). * *p* < 0.05, compared to the corresponding group.

TGF-β treatment led to an increase in expression of the mesenchymal cell marker α-SMA and a decrease in that of the epithelial cell marker E-cadherin (Figure 3A). Co-treatment with SB525334 reversed these changes. The levels of protein markers, including fibronectin, α-SMA, Snail and E-cadherin, as measured by Western blotting, showed the same trends as those from immunostaining (Figure 3B). Decreased Smad phosphorylation is known to contribute to decreased mRNA and protein levels of EMT inducers such as Snail [7]. In our study, the decreased Smad phosphorylation found was consistent with the decrease in protein and mRNA levels of Snail as its target (Figure 3B–D). TGF-β-treated HPMCs increased the synthesis of COL1A1, which was counteracted by co-treatment with SB525334 (Figure 3E,F).

### 3.3. Effects of SB525334 on an In Vivo Model

The body weight of mice from D0 to D21 was similar among the three treatment groups; however, it was lower in the PF alone group than in the CTL group at D28 (Supplementary Figure S2). Chow intake was also lower in the PF group than in the CTL group, but there were no significant differences in body weight or chow intake between the CTL and PF + SB groups (Table 1). The non-standardized β values (standard errors and *p* values) of chow intake for body weight at 1, 2, 3, or 4 weeks were 0.344 (0.114, *p* = 0.008), 0.291 (0.086, *p* = 0.003), 0.454 (0.102, *p* < 0.001) and 0.459 (0.070, *p* < 0.001), respectively. The peritoneal equilibrium test showed a decreased D/D0 glucose ratio in the PF group compared with that in the CTL group, but the ratio was recovered in the PF + SB group compared with that in the PF group (Supplementary Figure S3).

**Figure 4:**
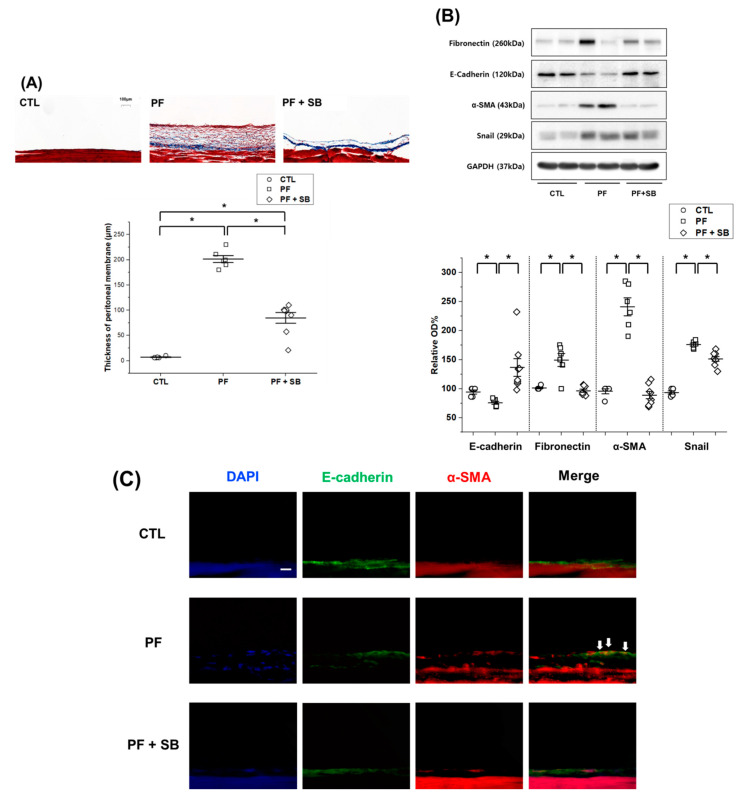

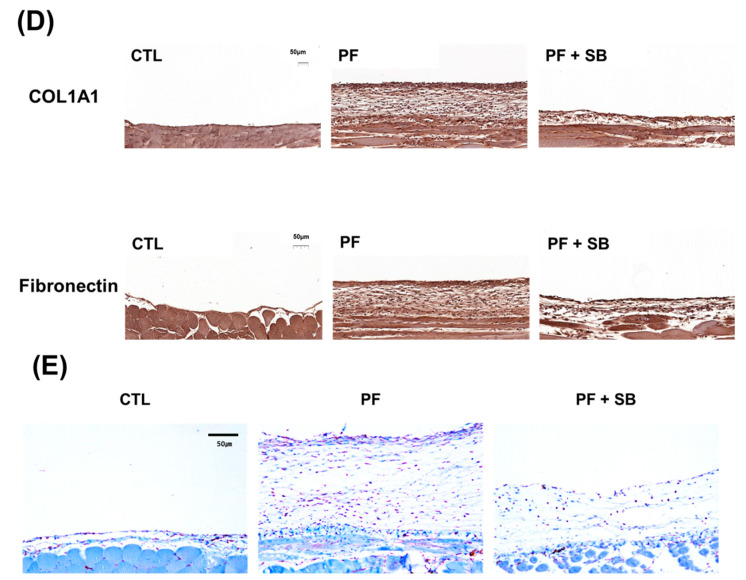
Morphological changes and Western blotting, immunofluorescence and immunohistochemical analyses of the parietal peritoneum. (**A**) Masson’s trichrome staining (100× magnification; scale bar, 100 μm). (**B**) Western blotting of epithelial or mesenchymal markers. (**C**) Immunofluorescence staining for E-cadherin (green) and α-SMA (red) with nuclear counterstaining (DAPI; blue) in the parietal peritoneum (400× magnification; scale bar, 20 μm). Cells positive for both E-cadherin and α-SMA are indicated by arrows. (**D**) Immunohistochemical staining for COL1A1 or fibronectin (200× magnification; scale bar, 50 μm). (**E**) Immunohistochemical staining for TGF-β (100× magnification; scale bar, 50 μm). CTL, control; PF, mice intraperitoneally injected with 0.1% chlorhexidine gluconate; PF + SB, PF with SB525334 administration by oral gavage; α-SMA, α-smooth muscle actin; OD, optical density; COL1A1, collagen type 1 alpha 1; TGF-β, transforming growth factor-beta 1. Thickness of the peritoneal membrane is expressed as mean ± standard error (*n* = 5 for CTL group, *n* = 6 for PF group, *n* = 8 for PF + SB group). * *p* < 0.05, compared to the corresponding group.

Trichrome staining showed that the thickness and collagen deposition of the parietal peritoneum in the PF group were increased, but these changes were attenuated by co-treatment with SB525334 (Figure 4A). In the PF group, the levels of fibronectin, α-SMA and Snail were increased, while those of E-cadherin were decreased, compared to those in the CTL group (Figure 4B). However, these changes were attenuated by treatment with SB525334. Immunostaining for DAPI, E-cadherin and α-SMA also showed similar trends to those observed with Western blotting (Figure 4C). The PF group included E-cadherin and α-SMA dual-positive cells, but the number of these cells decreased with SB525334 co-treatment. Immunohistochemical staining for COL1A1, fibronectin and TGF-β revealed increase in all factors in the PF group; however, these changes were attenuated by co-treatment with SB525334 (Figure 4D,E). In the liver, immunohistochemical staining with hematoxylin and eosin and trichrome for fibronectin and COL1A1 showed similar trends to that of the parietal peritoneum (Supplementary Figure S4).

## 4. Discussion

PF is a well-known, important pathologic change in patients with PD; previous studies have investigated various mechanisms and interventions for the attenuation of PF [2]. Peritoneal mesothelial cells can be damaged by various conditions, which leads to EMT of these cells as a classic pathogenesis of PF. The EMT of peritoneal mesothelial cells is associated with morphological and functional changes, such as loosening of intercellular tight junctions between mesothelial cells and invasion into the submesothelial space. Loosening of intercellular tight junctions is associated with increased vulnerability to bio-incompatible dialysate or inflammatory mediators and transformed/invaded cells lead to myofibroblast accumulation, submesothelial fibrosis and angiogenesis [3]. The TGF-β/Smad signaling pathway plays an important role in regulating these EMT changes in the peritoneal membrane. Previous studies have investigated the impact of various interventions, such as metformin, dexamethasone, tamoxifen, paricalcitol, or tranilast, on TGF-β-induced EMT and shown favorable results [12,13,14,19,20]. However, few are used in clinical settings, except for tamoxifen and/or steroids. Even tamoxifen and/or steroids are not strongly recommended for the prevention or treatment of PF, in clinical practice guidelines [4]. Most interventions consist of conservative care, such as modality switching or nutritional support. The unmet needs for prevention or treatment of peritoneal fibrosis necessitate further investigation of this process in peritoneal mesothelial cells.

SB525334 was originally characterized as a novel TβRI inhibitor and was firstly shown to attenuate fibrotic changes in an acute puromycin aminonucleoside nephropathy model [21]. SB525334 can inhibit the activity of TβRI and activin receptor-like kinase (ALK) 4; however, it does not inhibit other kinases, such as ALK3, or ALK6. In addition, inhibitory activity is greater against TβRI than ALK4. After the elucidation of TβRI inhibition by SB525334, the drug was evaluated using various pathological models, such as cancer or fibrosis, which are associated with the TGF-β signaling pathway. Higashiyama et al. investigated its protective effect in a bleomycin-induced pulmonary fibrosis model [22]. An in vitro study using rat chondrocytes showed that SB525334 decreased collagen production through downregulation of the extracellular signal-regulated kinase 1/2 (ERK1/2) and Smad signaling pathways [23]. A study investigating a Fabry disease model showed that SB525334 can attenuate the globotriaosylsphingosine and globotriaosylceramide-induced EMT of renal tubular cells, which results in protection from renal fibrosis [24]. Other studies have shown favorable results with SB525334 in liver treatments. Studies have investigated the anti-fibrotic effects of SB525334 using high-glucose and succinate-induced hepatic stellate cell activation or parasite infection models [25,26]. Huang et al. used a precision-cut liver tissue slice model that included all major cell types of liver parenchyma and showed anti-fibrotic effects of SB525334 in liver cells after bile duct ligation [27]. Other studies evaluated the influence of SB525334 on cancer models and revealed its inhibitory effects on the migration, invasion and EMT of cancer cells in the esophagus, liver, ovary and pancreas [8,9,10,11]. Furthermore, SB525334 attenuates the collagen production by reducing NADPH oxidase 4 in myoblasts [28].

Considering the importance of TGF-β-induced EMT in PF and the inhibitory effect of SB525334 on the EMT in various models, SB525334 may be useful in attenuating the fibrotic process in the PF model. In our study, we first induced EMT using TGF-β-treated HPMCs and found that these pathological changes were reversed by co-treatment with SB525334. These effects included morphological changes, cell migration and invasion and EMT markers. Finally, collagen synthesis was evaluated using immunofluorescence staining and qRT-PCR for COL1A1. Co-treatment with SB525334 attenuated all TGF-β-induced changes in EMT. We next performed an in vivo study using a CG induced PF model. CG injection was associated with an increase in PF and thickness, which functionally resulted in a decrease in the D/D0 ratio. Co-treatment with SB525334 attenuated these changes. The levels of EMT protein markers and immunohistochemical staining for fibrosis showed similar trends. Immunofluorescence staining for EMT markers showed induction of transformed cells with both epithelial and mesenchymal cell markers, which decreased upon co-treatment with SB525334. PF or thickness was also observed in the visceral peritoneum on the liver surface. These results were consistent with those from the parietal peritoneum. In addition, we performed immunohistochemical staining for TGF-β, which revealed that the CG-induced PF was associated with the TGF-β signaling pathway.

Our in vivo study revealed a positive association between chow intake and body weight. Intraperitoneally injectedCG, which results in peritoneal inflammation or fibrosis, was associated with a decrease in chow intake, which led to a decrease in body weight. Our in vivo study showed that, despite the lack of a significant difference in both chow intake and body weight between the PF and PF + SB groups, the trend showed that cotreatment with SB did not completely recover chow intake and body weight compared with those in the CTL group. This may be associated with two issues. First, the PF model using CG may be a very strong fibrosis model, which did not fully prevent injuries byCG, despite SB treatment. Second, PF by intraperitoneally injected CG can be developed through other pathways, such as NF-κB via advanced glycation end products, or IL-1β, or ERK 1/2 via IL-6, or angiotensin, beyond TGF-β and its signaling pathways [29,30,31]. SB525334 can attenuate TGF-β and its downstream pathways, but may not completely prevent changes through other pathways than TGF-β and its signaling pathways. The incomplete attenuation of peritoneal inflammation or fibrosis may not lead to full recovery of chow intake and body weight compared to those in the CTL group.

Our study has some limitations. First, in the in vitro study, EMT in HPMCs was induced by TGF-β, while EMT in the in vivo model was induced by CG injection. However, PF in PD is mainly caused by peritoneal dialysate with high glucose levels. In both in vitro and in vivo studies, the high glucose solution-induced EMT may be more equivalent to a human model of either TGF-β- or CG-induced EMT. In addition, the in vivo study was not performed under true uremic conditions. Second, human data for SB525334 are lacking owing to insufficient safety information, such as whether the drug is metabolized by the kidney. These can be important hurdles in developing clinical applications. However, as TβRII is a key mediator for EMT, targeted inhibition using SB525334 may have fewer side effects than other interventions, such as paricalcitol, metformin, or tranilast, which were originally developed for other purposes. Further preclinical studies regarding the safety of SB525334 should be considered to overcome these limitations.

## 5. Conclusions

SB525334, an orally available potent TβRI inhibitor, effectively attenuated the TGF-β-induced EMT in HPMCs. Cotreatment with SB525334 improved peritoneal thickness and fibrosis and recovered peritoneal membrane function in a PF mouse model.

## Figures and Tables

**Table 1:** Chow intake during the experimental period.

	CTL	PF	PF + SB
D1–D7 (g/week)	17.3 ± 0.6	14.6 ± 0.1 *	17.0 ± 0.4
D8–D14 (g/week)	17.7 ± 1.7	13.4 ± 0.3 *	16.0 ± 0.5
D15–D21 (g/week)	15.7 ± 1.9	13.4 ± 0.3	15.4 ± 1.2
D22–D28 (g/week)	16.8 ± 2.2	11.4 ± 0.1 *	13.5 ± 0.5

Comparison was performed using the Mann–Whitney U rank-sum test. Data are expressed as mean ± standard error (*n* = 5 for CTL group, *n* = 6 for PF group, *n* = 8 for PF + SB group). CTL, control; PF, mice intraperitoneally injected with 0.1% chlorhexidine gluconate; PF + SB, PF with SB525334 administration by oral gavage; D1, day 1; D7, day 7; D8, day 8; D14, day 14; D15, day 15; D21, day 21; D22, day 22; D28, day 28. * p < 0.05, compared to the CTL group.

## Data Availability

All data generated or analyzed during this study are included in the published article.

## Supplementary Materials

The following are available online at https://www.mdpi.com/article/10.3390/biomedicines9070839/s1, Figure S1. Cytotoxic effects of SB525334 on human peritoneal mesothelial cells (HPMCs). HPMCs were treated with SB525344 (0 μM, 0.1 μM, 1 μM, 10 μM, 100 μM and 200 μM) for 24, 48, or 72 h. SB525334 showed no significant toxicity up to 10 μM. Cell viability is expressed as mean ± standard error (n = 5 per group). * *p* < 0.05, compared to the corresponding group. Figure S2. Changes in body weight. CTL, control; PF, mice intraperitoneally injected with 0.1% chlorhexidine gluconate; PF + SB, PF with SB525334 administration by oral gavage. Data are expressed as mean ± standard error (n = 5 for CTL group, n = 6 for PF group, n = 8 for PF + SB group). * *p* < 0.05, compared to the CTL group. Figure S3. Peritoneal equilibrium test. CTL, control; PF, mice intraperitoneally injected with 0.1% chlorhexidine gluconate; PF + SB, PF with SB525334 administration by oral gavage; D/D0 glucose, the ratio of dialysate glucose level at 2 h after dialysate infusion per the level at 0 h. Data are expressed as mean ± standard error (n = 5 for CTL group, n = 6 for PF group, n = 8 for PF + SB group). * *p* < 0.05, compared to the CTL group. # *p* < 0.05, compared to the PF group. Figure S4. Morphologic changes in the liver peritoneum immunohistochemical staining with H&E, Masson’s trichrome, fibronectin, and COL1A1 (100× magnification; scale bar, 50 μm). CTL, control; PF, mice intraperitoneally injected with 0.1% chlorhexidine gluconate; PF + SB, PF with SB525334 administration by oral gavage; H&E, hematoxylin and eosin; COL1A1, collagen type 1 alpha 1.
