# Supplementary figures and images for: TGF-β1 Receptor Inhibitor SB525334 Attenuates the Epithelial to Mesenchymal Transition of Peritoneal Mesothelial Cells via the TGF-β1 Signaling Pathway
Source: Biomedicines. 2021 Jul 19;9(7):839. doi: 10.3390/biomedicines9070839 (PMC8301792; doi:10.3390/biomedicines9070839)

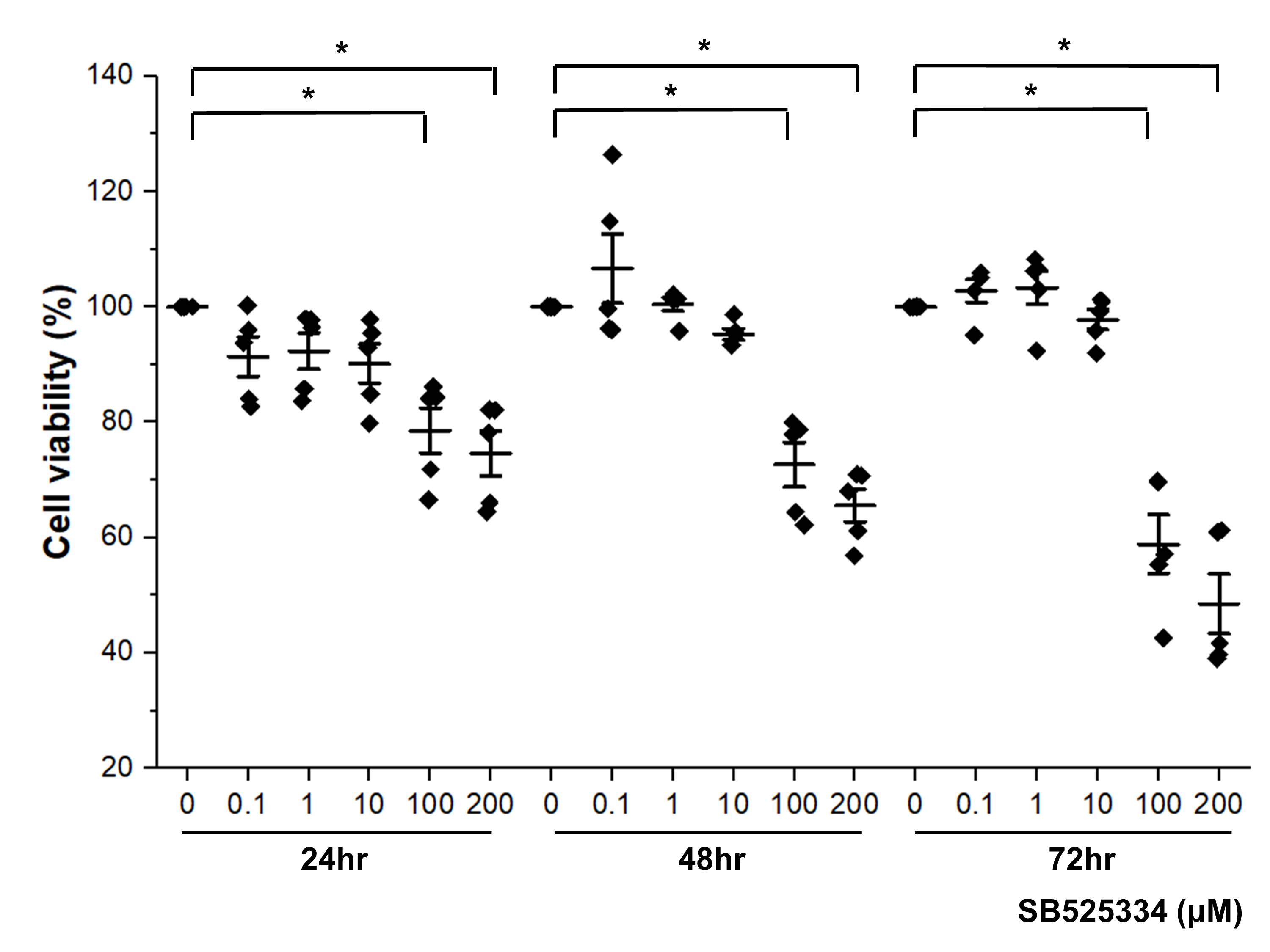

Supplement: Supplementary file 1 [file biomedicines-09-00839-s001.zip › Figure S1.jpg]

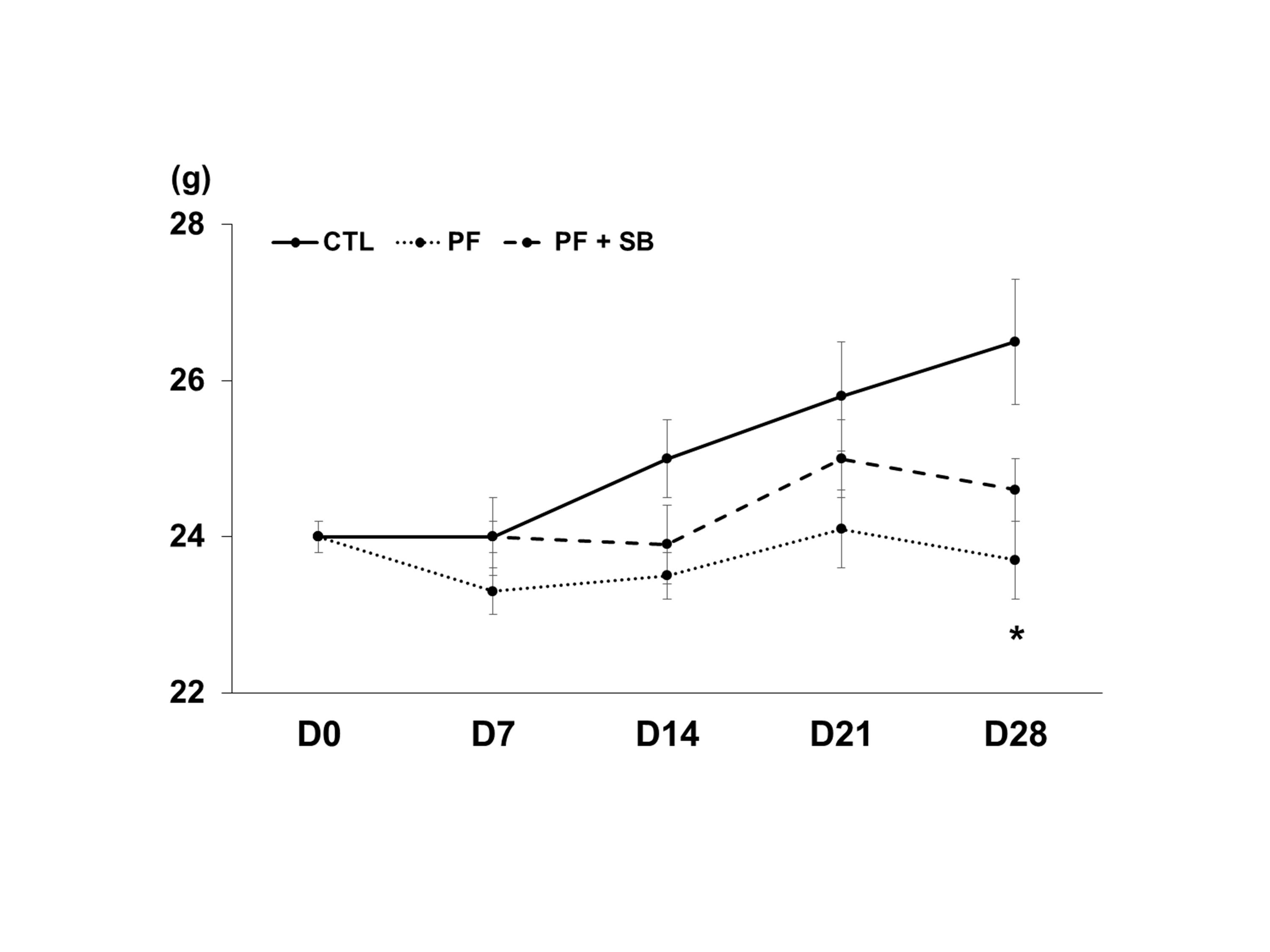

Supplement: Supplementary file 1 [file biomedicines-09-00839-s001.zip › Figure S2.jpg]

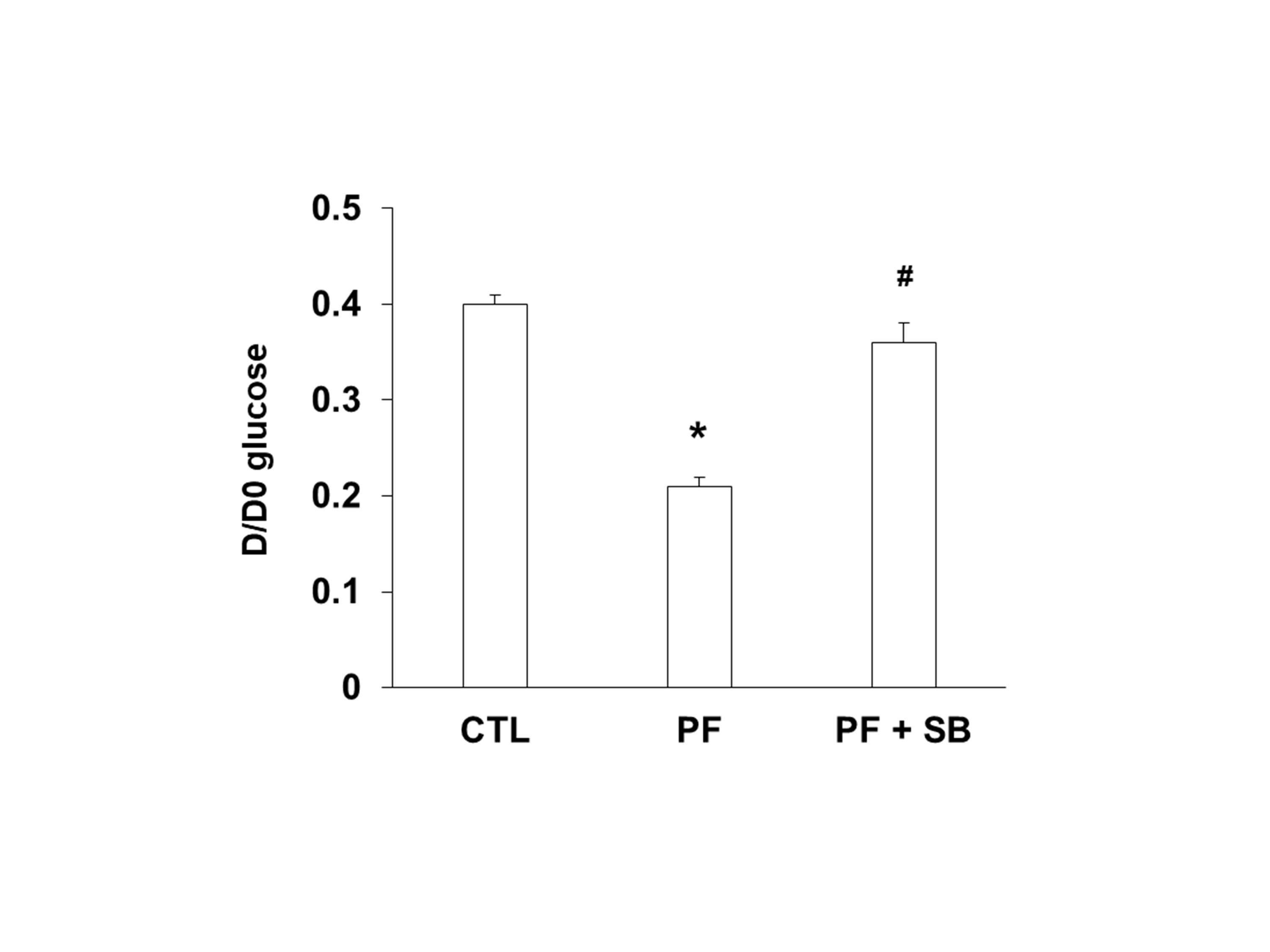

Supplement: Supplementary file 1 [file biomedicines-09-00839-s001.zip › Figure S3.jpg]

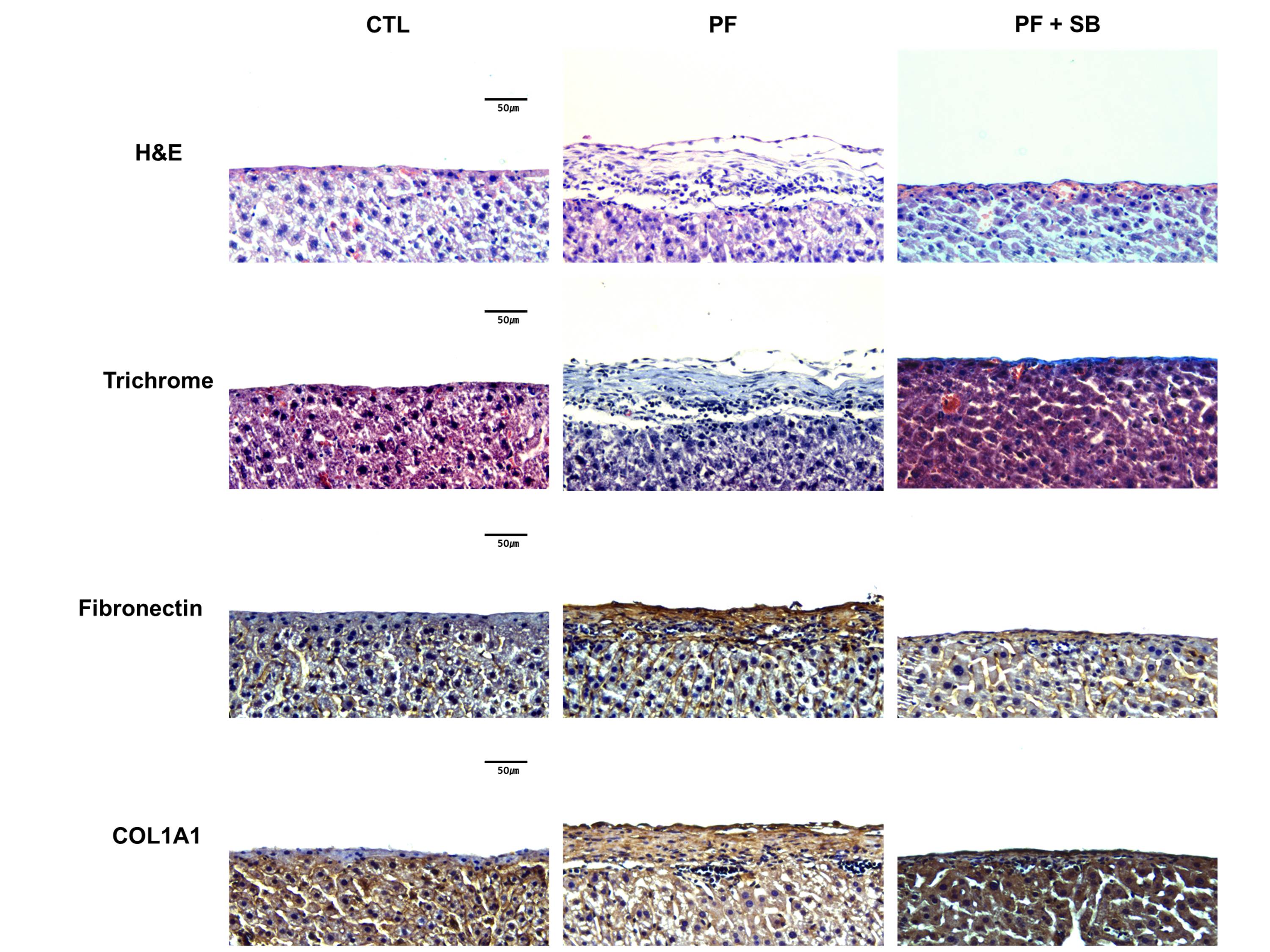

Supplement: Supplementary file 1 [file biomedicines-09-00839-s001.zip › Figure S4.jpg]
